# Demographic characteristics and comorbidities of patients with narcolepsy: a propensity-matched cohort study

**DOI:** 10.1093/sleepadvances/zpae067

**Published:** 2024-09-14

**Authors:** Melissa C Lipford, Wui Ip, Samir Awasthi, J Layne Moore, Maja Tippmann-Peikert, Shahir Asfahan, Praveen Kumar-M, Gajinder Pal Singh, Jennifer Gudeman

**Affiliations:** Mayo Clinic, Rochester, MN, USA; nference, Cambridge, MA, USA; nference, Cambridge, MA, USA; Mayo Clinic, Rochester, MN, USA; Mayo Clinic, Rochester, MN, USA; nference Labs, Bengaluru, India; nference Labs, Bengaluru, India; nference Labs, Bengaluru, India; Avadel Pharmaceuticals, Chesterfield, MO, USA

**Keywords:** narcolepsy, comorbidities, sleep disorders

## Abstract

**Study Objectives:**

Narcolepsy has a complex phenotype owing to differences in symptomatology, disease severity, and comorbidities. This is the first study to use aggregate electronic health record (EHR) data and natural language processing (NLP) algorithms to characterize the demographics and comorbidities of a large cohort of patients with narcolepsy.

**Methods:**

First-time Mayo Clinic patients (2000–2020) who had ≥1 narcolepsy-specific ICD-9/10 code and ≥1 disease-supportive statement in the clinical notes (identified using an NLP algorithm) were identified. A control cohort was propensity matched for birth year, age at first institutional encounter, sex, race, ethnicity, number of diagnosis codes, and mortality. Common comorbidities were compared and ranked between cohorts.

**Results:**

In the EHR database (*N* = 6 389 186 patients), 2057 patients with narcolepsy were identified (median age, 32 years; 59.6% female; 92.6% white; and 89.2% non-Hispanic) and propensity matched with a control cohort. Among the top 20 comorbidities occurring more frequently in the narcolepsy cohort compared with the control cohort (odds ratio [OR], 1.67–3.94; *p* < .001]) were sleep disorders (restless legs syndrome, obstructive sleep apnea, and insomnia), mood disorders (depression, dysthymia, and anxiety), and pain disorders (chronic pain syndrome, migraine, fibromyalgia, carpal tunnel syndrome, and myalgia). Other comorbidities significantly associated with narcolepsy (OR, 1.33–1.95) were irritable bowel syndrome (*p* < .001), asthma (*p* < .001), cervical spondylosis (*p* < .01), syncope (*p* < .01), and hypothyroidism (*p* < .05).

**Conclusions:**

This propensity-matched cohort study demonstrates increased psychiatric, sleep, and pain disorders in patients living with narcolepsy and challenges beyond narcolepsy-specific symptoms in this population. Understanding common narcolepsy-associated comorbidities may assist in tailoring treatment modalities.

**Statement of Significance**

Narcolepsy is a rare disease with variable symptoms and severity. Self-reported data have previously been used to characterize the phenotypes of narcolepsy, which may be complicated by reporter bias. Claims-based data have also been used, but availability is limited. Data from electronic health records (EHR) present a potentially more comprehensive view of patient information. In this study, data from EHRs of approximately 2000 patients with narcolepsy and 2000 patient controls confirmed the results of prior studies evaluating comorbidities and demographics of patients with narcolepsy compared to controls. Pain syndromes occurred at higher rates in the narcolepsy population, which has not been previously reported. The results of this study help to increase the understanding of challenges faced by patients with narcolepsy.

Narcolepsy is a rare chronic sleep disorder characterized by excessive daytime sleepiness that affects approximately 1 in 2000 people [1, 2]. Although peak symptom onset typically occurs in late adolescence or early adulthood, the average time from symptom onset to diagnosis is 5 to 15 years [1, 2]. Type 1 narcolepsy (NT1) is associated with cataplexy or sudden muscle weakness, typically in response to an emotion, whereas type 2 narcolepsy (NT2) is not associated with cataplexy; individuals with either type may also experience disturbed nighttime sleep, sleep paralysis, and hypnagogic or hypnopompic hallucinations [1]. In addition to this wide-ranging symptomatology, increased rates of psychiatric and sleep disorders have been reported in patients with narcolepsy [1, 3, 4]. Further complicating the care of this patient population is the chronic nature of narcolepsy; pharmacotherapy is generally lifelong and may need to be adjusted in line with potentially changing disease severity and comorbidities [1].

Previous studies examining the phenotype of narcolepsy have been limited by their design [3, 5]. For example, in their narcolepsy comorbidity study, Ohayon et al. relied on self-reporting for their data collection [3]. While useful, self-reported data can be hampered by difficulties in assessing the precision of patients’ recollection of symptoms and in reporting bias, as patients with narcolepsy may have greater awareness and more frequent reporting of their symptoms compared with the general population [3]. Ultimately, using self-reported data may potentially lead to inflated prevalence estimates [5].

The current study uses aggregate electronic health record (EHR) data obtained from the Mayo Clinic healthcare system to characterize the demographics and medical and psychiatric comorbidities of patients with narcolepsy without the use of self-reported data.

## Methods

### Mayo Clinic deidentified data and privacy

nference (Cambridge, MA, USA) is the exclusive data analytics engine for Mayo Clinic’s Clinical Data Analytics Platform. In partnership with Mayo Clinic, nference implemented a state-of-the-art deidentification tool that combines modern deep learning with rules and heuristics to transform each detected personally identifiable information instance into a suitable surrogate, effectively mitigating the risk of reidentification [6]. The research team only has access to deidentified data, pursuant to an expert determination in accordance with the Health Insurance Portability and Accountability Act Privacy Rule, representing the entire patient population at Mayo Clinic. These data only exist within Mayo Clinic’s secure cloud environment. To maintain full confidentiality, the results are only reported as aggregate statistics.

### Study design and participants

In 2021, an EHR-based search was conducted by nference in partnership with Mayo Clinic to identify patients with narcolepsy who presented for the first time at any Mayo Clinic campus between 2000 and 2020. Identified patients had ≥1 narcolepsy-specific International Classification of Disease version 9/10 code (ICD-9/10; 347.00, G47.419, 347, 347.01, G47.411, 347.10, G47.429, 347.0, G47.421, 347.11) and ≥1 disease-supportive statement (i.e. a diagnostic mention of narcolepsy) in their clinical notes. Qualifying statements were identified using a natural language processing (NLP) model. The model was developed to determine whether a patient has a condition mentioned in clinical notes, building upon the methodology described by Wagner et al. [7]. We queried sentences covering over 150 different diseases from various types of clinical notes, including outpatient clinic visit notes, hospital admission notes, and discharge summaries, within the Clinical Data Analytics Platform. These sentences were then labeled and used to fine-tune the pretrained SciBERT [8] sentence classification model to output one of the following labels: yes (confirmed diagnosis), no (ruled-out diagnosis), maybe (under consideration for differential diagnosis), and other (family history or insufficient context). The model was trained on 13 997 sentences from the Mayo Clinic EHR using an 85%:15% train:test split. It achieved an out-of-sample accuracy of 95.7%, with precision of 96.6%, and recall of 97.7% for the positive cases. In a separate test set specific to narcolepsy (*n* = 200), the model achieved an accuracy of 96.0%, with precision of 97.0%, and recall of 95.0% for the positive cases. The accuracy of the model was confirmed through a manual review of sentences extracted from clinical documentation at Mayo Clinic. If a sentence from the documentation contained confirmatory language that indicated a patient had a diagnosis of narcolepsy, it was considered a positive case.

A control cohort was created after propensity matching for birth year, age at first encounter at Mayo Clinic, sex, race, ethnicity, number of diagnosis code instances, and mortality as documented in the EHR to account for differences in demographics, care utilization, and severity of illness. Propensity score matching was employed to balance patient characteristics between groups using the nearest neighbor matching method. Propensity scores were estimated through a generalized linear model, ensuring comparability between the narcolepsy patient cohort and the control cohort. No imputation of missing values was performed as it was not necessary for the analysis. Comorbidities were defined based on ICD-9 and ICD-10 codes. Common comorbidities were identified by occurrences in the narcolepsy cohort compared with the control cohort. This study was approved by the Mayo Clinic Institutional Review Board (21-011867).

### Statistical analysis

Common comorbidities were compared between the narcolepsy patient cohort and the propensity-matched control cohort and then ranked by the odds ratio (OR) of their occurrences. Adjusted *p* values were calculated based on Bonferroni correction to account for multiple comparisons to reduce the risk of type 1 errors.

## Results

### Patient demographics

Of the 6 389 186 patients included in the EHR database whose records were assessed, 2057 patients with narcolepsy and 2057 propensity-matched control patients were identified (**Table 1**). Overall, demographics were similar between the cohorts (**Table 1**). The median age of participants at the first encounter at Mayo Clinic was 32 years in the narcolepsy cohort and 35 years in the control cohort. Nearly 60% of patients in each cohort were female, >90% were white, and >84% were non-Hispanic. The median number of diagnostic code instances was 224 in the narcolepsy cohort and 226 in the control cohort.

**Table 1.** Patient Demographics

Characteristic	Narcolepsy cohort *n* = 2057	Propensity-matched control cohort *n* = 2057
Median age at first encounter (IQR), y	32 (17–48)	35 (12–52)
Median birth year (IQR), y	1967 (1951–1983)	1963 (1948–1984)
Sex, *n* (%)		
Female	1227 (59.6)	1212 (58.9)
Male	830 (40.4)	845 (41.1)
Race, *n* (%)		
White	1905 (92.6)	1945 (94.6)
Black or African American	51 (2.5)	35 (1.7)
Unknown	18 (0.9)	11 (0.5)
Native American and Pacific Islander	15 (0.7)	12 (0.6)
Asian Far East	11 (0.5)	12 (0.6)
Other	57 (2.8)	42 (2.0)
Ethnicity, *n* (%)		
Not Hispanic or Latino	1834 (89.2)	1738 (84.5)
Hispanic or Latino	59 (2.9)	51 (2.5)
Unknown	138 (6.7)	244 (11.9)
Not disclosed	26 (1.3)	24 (1.2)
Deceased		
Yes	183 (8.9)	185 (9.0)
No	1874 (91.1)	1872 (91.0)
Median number of diagnostic codes (IQR)	224 (90–564)	226 (93–553)

IQR, interquartile range.

### Comorbidities

**Figure 1.**
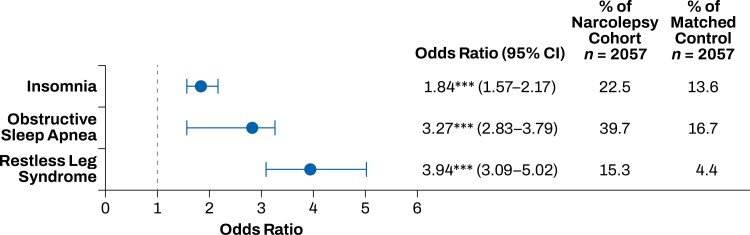
Sleep disorders comorbid with narcolepsy. Insomnia, obstructive sleep apnea, and restless legs syndrome had increased prevalence in the narcolepsy cohort compared with the propensity-matched control cohort. ***p < .001.

**Figure 2.**
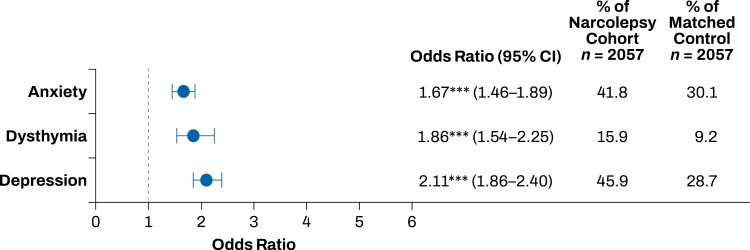
Mood disorders comorbid with narcolepsy. Anxiety, dysthymia, and depression had increased prevalence in the narcolepsy cohort compared with the propensity-matched control cohort. ***p < .001.

**Figure 3.**
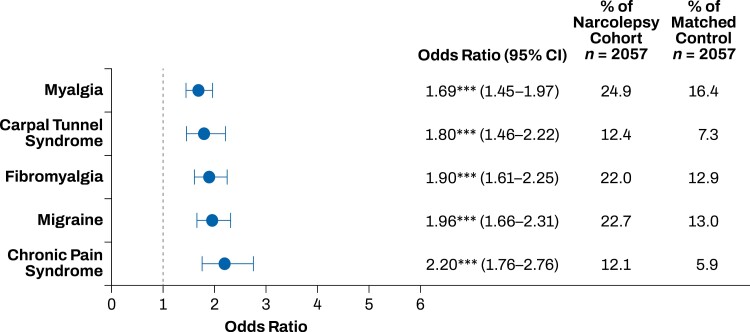
Pain disorders comorbid with narcolepsy. Myalgia, carpal tunnel syndrome, fibromyalgia, migraine, and chronic pain syndrome had increased prevalence in the narcolepsy cohort compared with the propensity-matched control cohort. ***p < .001.

**Figure 4.**
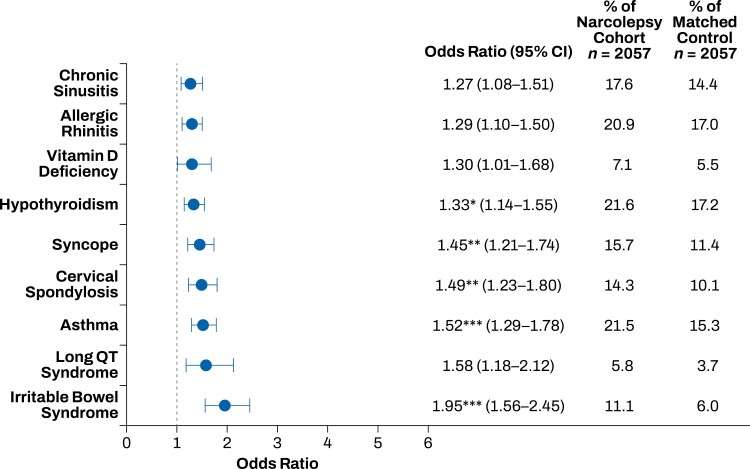
Other disorders comorbid with narcolepsy. Hypothyroidism, syncope, cervical spondylosis, asthma, and irritable bowel syndrome had significantly increased prevalence in the narcolepsy cohort compared with the propensity-matched control cohort. *p < .05; **p < .01; ***p < .001.

The top 20 comorbidities that occurred more frequently in the narcolepsy cohort compared with the matched control group included sleep, mood, and pain disorders. Sleep disorders that occurred more frequently in the narcolepsy cohort than in the control group included insomnia, obstructive sleep apnea, and restless legs syndrome (*p* < .001; **Figure 1**). Mood disorders that occurred more frequently in the narcolepsy cohort than in the control group included anxiety, dysthymia, and depression (*p* < .001; **Figure 2**). Pain disorders that occurred more frequently in the narcolepsy cohort than in the control group included myalgia, carpal tunnel syndrome, fibromyalgia, migraine, and chronic pain syndrome (*p* < .001; **Figure 3**). Other comorbidities that occurred significantly more frequently in the narcolepsy cohort than in the control group included hypothyroidism (*p* < .05), syncope (*p* < .01), cervical spondylosis (*p* < .01), asthma (*p* < .001), and irritable bowel syndrome (*p* < .001; **Figure 4**). There were no significant differences between the narcolepsy cohort and the control group for any additional clinically significant comorbidity beyond the top 20 identified (**Table 2**).

**Table 2.** Additional Disorders Comorbid With Narcolepsy

Comorbidity	Odds ratio (95% CI)	*p* value	Narcolepsy cohort, n (%) (*n* = 2057)	Propensity-matched control cohort, *n* (%) (*n* = 2057)
Obesity	1.35 (1.18 to 1.53)	< .001	738 (35.9)	604 (29.4)
Anemia	1.26 (1.04 to 1.52)	> .9	272 (13.2)	222 (10.8)
GERD	1.23 (1.08 to 1.40)	.109	734 (35.7)	639 (31.1)
Lumbosacral spondylosis	1.21 (1.03 to 1.42)	> .9	396 (19.3)	339 (16.5)
COPD	1.17 (0.94 to 1.45)	> .9	198 (9.6)	172 (8.4)
Diarrhea	1.15 (1.00 to 1.33)	> .9	520 (25.3)	467 (22.7)
Nicotine dependence	1.13 (0.97 to 1.33)	> .9	402 (19.5)	363 (17.6)
Constipation	1.13 (0.98 to 1.31)	> .9	462 (22.5)	419 (20.4)
Osteoarthritis	1.13 (0.99 to 1.29)	> .9	644 (31.3)	591 (28.7)
Diaphragmatic hernia	1.11 (0.91 to 1.35)	> .9	241 (11.7)	220 (10.7)
Myopia	1.11 (0.92 to 1.34)	> .9	266 (12.9)	243 (11.8)
Acute sinusitis	1.09 (0.92 to 1.28)	> .9	353 (17.2)	329 (16.0)
Nausea and vomiting	1.09 (0.95 to 1.25)	> .9	584 (28.4)	550 (26.7)
Polyneuropathy	1.08 (0.76 to 1.29)	> .9	66 (3.2)	61 (3.0)
Noninfective gastroenteritis and colitis	1.04 (0.87 to 1.25)	> .9	283 (13.8)	273 (13.3)
Hypertension	1.04 (0.92 to 1.18)	> .9	875 (42.5)	855 (41.6)
Coronary artery disease	1.03 (0.83 to 1.29)	> .9	172 (8.4)	167 (8.1)
Mitral valve disorder	1.03 (0.84 to 1.26)	> .9	213 (10.4)	207 (10.1)
Arrhythmia	1.02 (0.90 to 1.18)	> .9	666 (32.4)	657 (31.9)
Presbyopia	1.00 (0.83 to 1.20)	> .9	266 (12.9)	266 (12.9)
Urinary tract infection	0.97 (0.84 to 1.12)	> .9	488 (23.7)	498 (24.2)
Type 2 diabetes mellitus	0.95 (0.81 to 1.12)	> .9	349 (17.0)	364 (17.7)
Heart failure	0.95 (0.78 to 1.15)	> .9	224 (10.9)	235 (11.4)
Hyperlipidemia	0.93 (0.82 to 1.05)	> .9	846 (41.1)	883 (42.9)
Tricuspid valve disorder	0.91 (0.60 to 1.39)	> .9	43 (2.1)	47 (2.3)
Osteoporosis	0.90 (0.74 to 1.10)	> .9	205 (10.0)	225 (10.9)
Acute upper respiratory infection	0.84 (0.74 to 0.95)	.3	858 (41.7)	948 (46.1)
Contact dermatitis	0.84 (0.72 to 0.99)	> .9	365 (17.7)	419 (20.4)
Chronic kidney disease	0.84 (0.70 to 1.0)	> .9	250 (12.2)	292 (14.2)
Pneumonia	0.84 (0.68 to 1.03)	> .9	178 (8.7)	209 (10.2)
Seborrheic keratosis	0.83 (0.70 to 0.99)	> .9	292 (14.2)	341 (16.6)
Atrial fibrillation	0.82 (0.66 to 1.02)	> .9	170 (8.3)	203 (9.9)
Colonic polyp	0.80 (0.68 to 0.95)	.569	315 (15.3)	378 (18.4)
Acute kidney failure	0.79 (0.64 to 0.97)	> .9	182 (8.8)	226 (11.0)
Diverticulosis	0.78 (0.66 to 0.92)	.212	302 (14.7)	372 (18.1)
Pleural effusion	0.75 (0.62 to 0.92)	.365	191 (9.3)	246 (12.0)
Otitis media	0.64 (0.54 to 0.76)	< .001	262 (12.7)	383 (18.6)
Actinic keratosis	0.73 (0.60 to 0.89)	.134	194 (9.4)	256 (12.4)

COPD, chronic obstructive pulmonary disease; GERD, gastroesophageal reflux disease.

## Discussion

This propensity-matched cohort study affirmed prior studies that demonstrated increased psychiatric and sleep disorders in patients with narcolepsy [3, 5, 9] and underscores the complexity of this patient population. Among the top 20 comorbidities that were increased in the narcolepsy group, 5 disorders (25%) were related to various types of pain, with chronic pain syndrome more than twice as likely to occur in the narcolepsy cohort than in the matched cohort. To the best of our knowledge, pain syndromes have not been previously identified as having increased comorbidity among patients with narcolepsy using a large real-world cohort. Impaired nocturnal sleep, which is common among patients with narcolepsy, is associated with new incidents of chronic pain and exacerbation of existing chronic pain and may contribute to the high incidence of pain-related comorbidities in this cohort [10]. Similarly to how impaired nocturnal sleep exacerbates existing chronic pain, pain also disturbs sleep, and this bidirectional relationship suggests that improving sleep may alleviate pain symptoms [11]. Recognizing common comorbid conditions, such as psychiatric, sleep, and pain disorders, in patients with narcolepsy may help clinicians with appropriate management [3, 4].

It is noteworthy that cardiovascular diseases (CVD), including hypertension, were not among the top 20 comorbidities among >2000 patients with narcolepsy compared with an EHR-based, propensity-matched, non-narcolepsy cohort. While the data generated through this retrospective EHR review may seem to contrast recent publications showing an increased risk of CVD in patients with narcolepsy [12, 13], it is important to consider the context and different aims of prior research. In the present study, the aim was to identify the top 20 most prevalent comorbidities, agnostic to a specific class of disease, among >2000 patients with narcolepsy compared with a matched cohort of patients without narcolepsy. Within this context of most common comorbidities, CVD was not shown in the top 20. A similar agnostic approach was taken in the initial impact of narcolepsy disease (BOND) study, which demonstrated other significantly increased comorbidities in the cohort of patients with narcolepsy [4]. The highest ORs demonstrating comorbidity prevalence were observed for mental illness (OR 3.8), respiratory (OR 3.7), neurological (other than narcolepsy; OR 3.7), and musculoskeletal disorders (OR 3.5). Additional conditions included endocrine/nutritional/metabolic diseases and immunity disorders (OR 2.8), diseases of the digestive (OR 2.7), circulatory system (OR 2.6), and injury, and poisoning (OR 2.4). Results of the subsequent CV-BOND study solely focused on new-onset CVD; patients in the narcolepsy cohort experienced statistically significant increases in event incidence rates compared to the matched control cohort [12]. Claims databases provide very large datasets and may be instructive. However, as cited by Barateau and Dauvilliers [14], the CV-BOND analysis was limited by a lack of information regarding the treatments used by the narcolepsy cohort, the unavailability of body mass index data, and the inclusion of more patients with NT2 than NT1. Furthermore, CV-BOND does not provide insight into the most prevalent conditions increased in a narcolepsy cohort compared to those without narcolepsy, as both the present research and BOND provided.

In a community-based cohort of patients with narcolepsy, the highest ORs demonstrating comorbidity prevalence both at diagnosis and at the end of an approximately decade-long observation period were for obstructive sleep apnea (OR, 69.3 and 13.6, respectively), chronic low back pain (OR, 5.5 and 2.6), and depression (OR, 4.9 and 3.8) [15]. Additionally, certain comorbidities (e.g. anxiety disorder, thyroid disease, hypertension, and hyperlipidemia) were increased at initial narcolepsy diagnosis but became similar in prevalence to the age-matched control cohort over time. The change in comorbidity prevalence over time is relevant given that narcolepsy is a chronic, lifelong disease. This cohort also demonstrated an elevated prevalence of obesity at diagnosis and at the end of the observation period (OR, 2.3 and 2.1) [15]. In our analysis, a statistically significantly higher rate of obesity (*p* < .001) was observed in the narcolepsy cohort compared with the control cohort. The prevalence of eating disorders and obesity in people with narcolepsy has been the subject of recent literature; however, the findings have been conflicting [16, 17]. Results of a cross-sectional study conducted by Baldini et al. [16] indicated that eating disorders were more prevalent in study participants with NT1 compared with healthy controls. Conversely, Dahmen et al. [17] did not find an increased prevalence of eating disorders among participants with narcolepsy compared with healthy control participants. In our study, eating disorders were not identified as a comorbidity among the narcolepsy cohort. While the etiology of obesity in the present study is unknown, further research into the association between obesity and eating disorders in people with narcolepsy may be warranted.

Understanding narcolepsy remains challenging owing to multiple factors, including the complex phenotype and the relative rarity (i.e. 1 in 2000 Americans) of the disease [2]. The path to receiving a narcolepsy diagnosis is long, and it often includes misdiagnoses and/or a “missed” narcolepsy diagnosis; that is, a patient may be accurately diagnosed with depression and/or obstructive sleep apnea, but the additional diagnosis of narcolepsy is not identified until a later time [2, 3]. Conversely, once a patient is diagnosed with narcolepsy, it remains essential to ensure comorbidities have been appropriately diagnosed and are being addressed within a comprehensive management plan [18, 19].

A limitation of using diagnostic codes is that all relevant codes must be recorded for each patient to ensure accurate record keeping, thus reducing the ability to differentiate between patients with the distinct conditions NT1 and NT2 in this study. Patients may be initially diagnosed with NT2; if cataplexy (particularly when subtle) is later identified, the diagnosis would be changed to NT1 [20]. Thus, the results of the current study are generalizable to the general population of patients with narcolepsy without distinction for different types (NT1 vs NT2). Although diagnosis codes are imperfect, the use of NLP and ICD-9/10 codes together avoided the limitations associated with patient self-reporting in other studies by eliminating reliance on patient recall, thereby decreasing reporting bias [3, 5]. Propensity matching was used for birth year, age at first encounter at a Mayo Clinic, sex, race, ethnicity, number of diagnosis code instances, and mortality as documented in the EHR; however, unidentified differences in the narcolepsy and non-narcolepsy cohorts may exist. EHR utilization may provide a more well-balanced comparison than claims data [21], which have been used in the past to compare healthcare utilization patterns among patients with narcolepsy [22]. Last, this analysis only used records from Mayo Clinic. Although Mayo Clinic has locations at various sites in the United States, this cohort may not be representative of the entire narcolepsy population, particularly because Mayo Clinic is often a referral center for more challenging cases. A final and important limitation is the lack of racial and ethnic diversity within the study cohort.

## Conclusions

This research increases the understanding of common comorbidities in narcolepsy without the limitation of patient self-reporting or claims data. A greater understanding of common narcolepsy-associated comorbidities may help optimize treatment efficacy and result in a better comprehension of the medical and psychiatric challenges of patients with narcolepsy.

## Data Availability

This study involves the analysis of deidentified Electronic Health Record (EHR) data via the nference Analytics Platform. Data shown and reported in this manuscript have been extracted from this environment using an established protocol for data extraction, aimed at preserving patient privacy. The data have been deidentified pursuant to an expert determination in accordance with the HIPAA Privacy Rule. Any data beyond what is reported in the manuscript, including but not limited to the raw EHR data, cannot be shared or released due to the parameters of the expert determination to maintain the data deidentification. Contact corresponding authors for additional details regarding the nference nSights Analytics Platform.
